# A paclitaxel prodrug nanoparticles with glutathion/reactive oxygen species dual‐responsive and CD206 targeting to improve the anti‐tumour effect

**DOI:** 10.1049/nbt2.12119

**Published:** 2023-04-13

**Authors:** Changhai Wang, Yuwen Jiao, Xinyu Zhang, Mingxue Guo, Qing Zhang, Wenjun Hu, Shuang Dong, Tangthianchaichana Jakkree, Yang Lu, Jinling Wang

**Affiliations:** ^1^ Department of Beijing University of Chinese Medicine Beijing China; ^2^ Thammasat University Pathum Thani Thailand

**Keywords:** cellular effects of radiation, drugs, drug delivery systems, nanoparticle, tumours

## Abstract

As a first‐line anticancer drug, paclitaxel has shortcomings, such as poor solubility and lack of tumour cell selectivity, which limit its further applications in clinical practice. Therefore, the authors aimed to utilise the characteristics of prodrug and nanotechnology to prepare a reactive oxygen species (ROS) and GSH dual‐responsive targeted tumour prodrug nanoparticle Man‐PEG‐SS‐PLGA/ProPTX to improve the clinical application status of paclitaxel limitation. The characterisation of Man‐PEG‐SS‐PLGA/ProPTX was carried out through preparation. The cytotoxicity of nanoparticles on tumour cells and the effect on apoptosis of tumour cells were investigated by cytotoxicity assay and flow cytometry analysis. The ROS responsiveness of nanoparticles was investigated by detecting the ROS level of tumour cells. The tumour cell selectivity of the nanoparticles was further investigated by receptor affinity assay and cell uptake assay. The particle size of Man‐PEG‐SS‐PLGA/ProPTX was (132.90 ± 1.81) nm, the dispersion coefficient Polymer dispersity index was 0.13 ± 0.03, and the Zeta potential was (−8.65 ± 0.50) mV. The encapsulation rate was 95.46 ± 2.31% and the drug load was 13.65 ± 2.31%. The nanoparticles could significantly inhibit the proliferation and promote apoptosis of MCF‐7, HepG2, and MDA‐MB‐231 tumour cells. It has good ROS response characteristics and targeting. The targeted uptake mechanism is energy‐dependent and endocytosis is mediated by non‐clathrin, non‐caveolin, lipid raft/caveolin, and cyclooxygenase (COX)/caveolin with a certain concentration dependence and time dependence. Man‐PEG‐SS‐PLGA/ProPTX is a tumour microenvironment‐responsive nanoparticle that can actively target tumour cells. It restricts the release of PTX in normal tissues, enhances its selectivity to tumour cells, and has significant antitumour activity, which is expected to solve the current limitations of PTX use.

## Graphical abstract

(1). We take advantage of prodrug characteristics and nanotechnology to prepare an ROS and GSH dual‐responsive targeted tumour prodrug nanoparticle Man‐PEG‐ss‐PLGA/pro‐PTX. (2). Man‐PEG‐SS‐PLGA/ProPTX restricts the release of PTX in normal tissues, enhances its selectivity to tumour cells, and has significant antitumour activity, which is expected to solve the current limitations of PTX use.

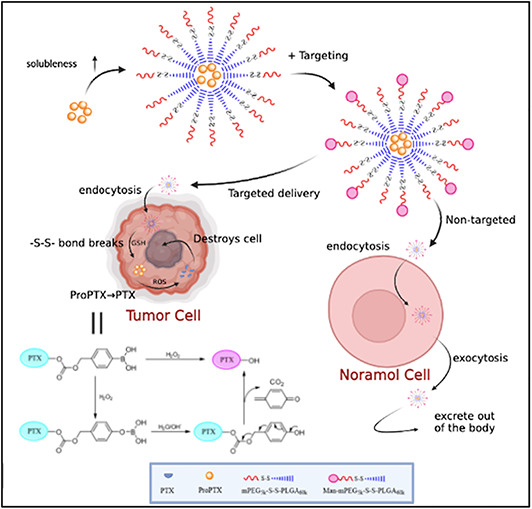

## 1 INTRODUCTION

Paclitaxel (PTX) is one of the most excellent chemotherapeutic drugs. It has been widely used in the first‐line treatment of breast cancer [1], ovarian cancer [2], lung cancer [3], and head and neck cancer [4]. However, poor solubility and lack of tumour cell selectivity are two major difficulties in the clinical application of PTX. At present, the problem of poor solubility can be solved to some extent by dissolving PTX in the mixture of polyoxyethylene castor oil and absolute ethanol. Unfortunately, polyoxyethylene castor oil can cause serious side effects [5]. In addition, the lack of tumour cell selectivity after drug entry into the body will also cause serious adverse reactions [6], which limits the clinical application of PTX to a large extent.

Precursor drugs, also known as prodrugs, are chemically modified to make their parent drugs have no biological activity or low activity in vitro and play a role in the specific environment in vivo by structural dissociation and release of the parent drug. The tumour microenvironment is a special internal environment existing in the tumour site, in which the tumour cells have high concentrations of reduced glutathion (GSH) and high levels of reactive oxygen species (ROS) [6]. Therefore, intelligent responsive drug delivery systems can be designed for tumour microenvironment characteristics to enhance efficacy and reduce toxicity. A ROS‐sensitive prodrug for paclitaxel was synthesised by phenolate quinol ester (PBA). It can release active parent drug PTX under the stimulation of a high level of ROS in tumour cells to exert an anti‐tumour effect while remaining inactive prodrug in non‐tumour sites. This prodrug is expected to avoid the release of drugs in normal tissues and can reduce the adverse reactions and side effects of PTX [7]. However, it still has shortcomings, such as poor solubility, fast metabolism in vivo circulation, and does not solve the problem of lack of selectivity of drugs on tumour cells.

The nano‐drug delivery system encapsulates chemotherapeutic drugs in nano‐scale carrier materials, which can significantly improve the solubility of insoluble drugs and prolong the systemic circulation time of drugs, thus improving drug efficacy [8]. On the other hand, the nano‐drug delivery system would also accumulate in the tumour site through the Enhanced permeability and retention effect (EPR), reducing the distribution of the drug in the normal tissue and reducing the adverse reactions.

In conclusion, prodrug nanoparticles with GSH/ROS dual‐responsive are of great significance in improving the effect of PTX cancer treatment and alleviating side effects [9, 10]. After modifying the tumour targeting structure, its selectivity to tumour cells will be further enhanced [11]. The mannose receptor CD206 is a transmembrane glycoprotein. It is highly expressed on a variety of tumour cells and the surface of tissue macrophages, dendritic cells, and lymphocytes in tumour sites [12]. It can regulate primary tumour growth, anti‐tumour adaptive immune response, and tumour angiogenesis and promote tumour spread and metastasis [13]. Therefore, CD206 can be modified in nano delivery systems to give an active targeting function.

In this study, the GSH‐sensitive amphiphilic compound Man‐PEG_5K_‐SS‐PLGA_60K_ was used as a polymer material to encapsulate ROS‐sensitive phenylboronic acid‐paclitaxel prodrug (ProPTX) synthesised in our group. The mannose, a structure targeting the CD206 target, is then modified on the surface of the package. Finally, a tumour microenvironment‐responsive targeted prodrug nanoparticle, Man‐ProPTX‐SS‐NPs, was prepared. The PLGA side chain of Man‐PEG_5K_‐SS‐PLGA_60K_ can stably and efficiently carry ProPTX and has good biocompatibility and biodegradation. Man‐PEG_5K_‐SS‐PLGA_60K_ has good biocompatibility and biodegradability. Its PLGA side chain can stably and efficiently load ProPTX. The PEG side chain can extend outside to produce a shielding effect, which can inhibit the adsorption of nano‐drug protein and prevent rapid elimination, thus prolonging the drug circulation time in vivo. In addition, surface modification of the Mannose targeting structure combined with the EPR effect can efficiently deliver nanomaterials to the tumour site and accumulate. This CD206‐targeted nanoparticle combined with ROS and GSH dual response can effectively solve the problem of poor solubility of PTX and lack of selectivity for tumour cells.

Based on this, we investigated the optimal preparation process of prodrug nanoparticles and characterised them by using particle size and Polymer dispersity index as indicators. In addition, the in vitro responsive release ability and toxicity of the nanoparticles prepared by the optimal process were further investigated to prepare high tumour‐specific anti‐tumour drugs of paclitaxel.

## 2 MATERIALS

### 2.1 Chemicals and reagents

Paclitaxel prodrug (ProPTX, 98%, self‐synthesis), Paclitaxel (PTX, 98%, Shanghai Aladdin Biochemical Technology Co., LTD.), Man‐PEG_5k_‐PLGA_60k_ (75:25, Jinan Dagang Biotechnology Co., LTD.), Man‐PEG_5k_‐SS‐PLGA_60k_, DSPE‐PEG_5k_, DSPE‐PEG_5k_‐mannose (99%, Xi ‘an Ruixi Biological Co., LTD.); RPMI1640 medium (Corning Company, USA); GIBCO Australian Foetal Bovine Serum (Beijing Benovit Biotechnology Co., LTD.); Trypsin (Nanjing Kaiji Biotechnology Co., LTD.); Tetramethyl azolium blue (MTT, Sigma, USA); Coumarin‐6 (Sigma, USA); BCA protein Quantification Kit (Kangwei Century Biotechnology Co., LTD.); Tritonx‐100 (Beijing Apisi Biotechnology Co., LTD.); DAPI (Sigma Company, USA), other reagents were analytically or chromatographically pure.

Human breast cancer cell MCF‐7 (Nanjing KGI Biotechnology Co., LTD.), Human hepatocellular carcinoma cell line HepG2 (Cell Resource Centre, Institute of Basic Medical Sciences, Chinese Academy of Medical Sciences and Peking Union Medical College), Mda‐mb‐231 (Nanjing Kaiji Biotechnology Co., LTD.)

### 2.2 Instrumentation

Electronic balance (BS224S, Sartorius, Germany); Laser particle size analyser (Nano‐ZS90, Malvern Instruments LTD.); R‐200 Rotary evaporator (BUCHI, Switzerland); Shimadzu LC‐20ADXR High Performance liquid chromatograph (Shimadzu Company, Japan); C18 column (5 μm, 4.6 × 250 mm, Agilent Technologies); Multiskan GO full‐wavelength scanning microplate reader (Thermo, USA); Ultrasonic crushing instrument (Beijing Zhongke Kor Instrument Co., LTD.); Fluorescence inverted microscope IX71 (Olympus, Japan); OptiMair Ultra Clean Workstations (ESCO, Singapore); Zeiss LSM 800 laser confocal microscope (confocal laser microscopy (CLSM), Beijing Opotong Optical Technology Co., LTD.); Multiskan GO full‐wavelength scanning microplate reader (Thermo, USA); Me2.04 millionth electronic balance (Mettler Toledo Instruments (Shanghai) Co., LTD.); Df‐101s collector thermostatic magnetic stirrer (Gongyi Yingyu Yuhua Instrument Factory); Flow cytometry (Becton Dickinson, USA).

## 3 METHOD

### 3.1 Preparation of man‐PEG‐SS‐PLGA paclitaxel prodrug nanoparticles

1 mg ProPTX, 5 mg Man‐PEG_5k_‐SS‐PLGA_60k_, and 1 mg DSPE‐PEG_5K_‐Mannose were weighed and dissolved in 1 mL acetone. Under the condition of ice bath ultrasound (8 min, 150 W), the obtained solution was added drop by drop to 10 mL of distilled water containing 0.02% TPGS. After an ultrasound, the prodrug nanoparticles Man‐ProPTX‐SS‐NPs were obtained by drying acetone at room temperature and passing through a 0.45 μm filter membrane.

### 3.2 Particle size and zeta potential

The particle size and potential of ProPTX‐SS‐NPs were determined by the Malvern particle size analyser at 25°C, and the results were averaged after three parallel operations.

### 3.3 Stability of proPTX colloid

Using PBS (pH 7.4) containing 10% foetal bovine serum as an incubation medium, the colloidal stability of prodrug nanoparticles was investigated by analysing the particle size changes during incubation. After mixing the nanoparticle solution with the incubation medium (1:5, v/v), the solution was placed in a 37°C thermostatic oscillator at a speed of 100 rpm and incubated for 24 h. Samples were taken at 0, 2, 4, 6, 8, 10, 12, and 24 h to determine the particle size of the nanoparticle.

### 3.4 Encapsulation rate and drug loading

An amount of 100 μL of the precursor nanoparticle solution was diluted by adding 900 μL of acetonitrile, vortexed for 10 min, centrifuged at 12,000 rpm for 10 min, and 800 μL of the supernatant was taken for bottling. The ProPTX content in prodrug nanoparticles was determined by the HPLC method established in Section 3.1. The encapsulation rate (EE) and drug load (DL) of the drug were calculated according to the following formula:

Encapsulationefficiency(EE%)=drugcontentinnanoparticles/totaldrugweight×100%

Drugloading(DL%)=drugcontentinfreeze−driednanoparticles/weightoffreeze−driednanoparticles×100%

### 3.5 Cytotoxicity

MTT assay was used to investigate the growth inhibition of blank nanoparticles, PTX, ProPTX, and prodrug nanoparticles on MDA‐MB‐231 cells. The cells with a good growth status in the logarithmic growth stage were seeded into 96‐well plates at 5 × 10^4^ cells/mL and incubated in 5%CO_2_ for 24 h at 37°C. The cells were treated with different drug solutions for 24 h, and the control wells were treated with a 100 μL complete medium. After drug treatment, the liquid in each well was aspirated and 150 μL of DMSO was added. Then, the light was avoided and shaken for 10 min to dissolve the blue‐purple crystal. The absorbance values of each well were measured at 570 nm using a full‐wavelength scanning microplate reader.

$$Inhibition\;Rate\;\left(\%\right)=1–A_{formulations}\;/A_{control}$$

A:formulationAbsorbancevalueofdosinghole;A:controlAbsorbancevalueofcontrolhole

### 3.6 Apoptosis

#### 3.6.1 Cell morphology was observed by an inverted microscope

MDA‐MB‐231 cells were spread in a 6‐well plate at a density of 1.5 × 10^5^ cells/mL. After the cells were attached to the wall, the culture medium was discarded. And, 2 mL PTX, ProPTX, ProPTX‐NPs, ProPTX‐SS‐NPs, and Man‐ProPTX‐SS‐NPs were added, respectively (the final concentration of paclitaxel was 10 μm). After 48 h of incubation, the culture medium was discarded. The molecular layer of cells was washed twice with PBS at 4°C. The cells were fixed with 4% paraformaldehyde for 20 min and washed with PBS 3 times, 1 min each. Under the condition of avoiding light, 350 μL DAPI solution (5 μg/mL) was added and incubated for 15–20 min. The DAPI solution was discarded and washed with PBS 3 times, 1 min each time, and then the cell morphology was observed under a laser confocal microscope.

#### 3.6.2 Cell apoptosis was analysed by flow cytometry

Normal growing MDA‐MB‐231 cells were taken and spread in a 6‐well plate at the density of 1 × 10^5^ cells/mL. After the cells were attached to the wall, the culture medium was discarded. PTX, ProPTX, ProPTX‐NPs, ProPTX‐SS‐NPs, and Man‐ProPTX‐SS‐NPs were added and incubated for 48 h. The drug solution was discarded. The cell monolayer was washed with PBS buffer 4°C for 3 times and then digested with trypsin. The cells were neutralised with the PBS solution containing 10% FBS, centrifuged at 800 rpm for 5 min, the supernatant was discarded, and the cells were washed twice with Binding Buffer. The cells were resuspended with 100 μL of 1 × Binding Buffer, then 5 μL of AnnexinV‐FITC was added, and the Staining was placed in the dark for 10 min. 5 μL PI solution was added to the cell suspension and the reaction was performed at 25℃ and in the dark for 5 min; 100 μL of 1 × Binding Buffer was added and gently mixed. Within 1 h, apoptosis was analysed by flow cytometry.

### 3.7 Determination of ROS levels

To investigate the sensitivity of H_2_O_2_, MDA‐MB‐231 cells were seeded in a six‐well plate at a concentration of 2 × 10^5^ cells/well and cultured for 24 h. PTX, ProPTX, Blank PP‐NPs, and PTX physical mixture, Blank PP‐NPs and ProPTX physical mixture, PTX‐NPs, ProPTX‐NPs, ProPTX‐SS‐NPs, and Man‐ProPTX‐SS‐NPs were added, respectively (the final concentration of paclitaxel was 10 μm). Then, 100 μL EDTA‐free trypsin was added to each well for digestion, which was neutralised with the PBS solution containing 10% FBS, and transferred to a 1.5 mL centrifuge tube, centrifuged at 350 g for 5 min. The cells were washed twice with PBS, then an 800 μL serum‐free medium containing 10 μm DCFH‐Da was added, and the cells were incubated at 37°C for 30 min. The DCFH‐DA that did not enter the cells was removed by washing with PBS three times. Finally, 300 μL PBS was added to resuspend the cells. The level of ROS in 10,000 cells of each sample was quantified by flow cytometry.

### 3.8 Cell uptake

#### 3.8.1 The uptake of nanoparticles in MDA‐MB‐231 cells was qualitatively investigated

The non‐fluorescent PTX was replaced with strongly fluorescent coumarin‐6 (C6), and each nanoparticle was prepared by the same method as described in Section 2.1. MDA‐MB‐231 cells were seeded uniformly in a 30 mm^2^ laser confocal dish at a density of 1 × 10^5^ cells/well and incubated in a CO_2_ incubator for 24 h. After the cells were attached to the wall, the cell monolayer was washed twice with HBSS, 1.5 mL of HBSS solution was added to the culture dish, and the culture was incubated in the incubator for 30 min, and then the HBSS solution was discarded. 1.5 mL of C6‐Sol and different C6 drug‐loaded nanoparticles (C6‐NPs, C6‐SS‐NPs, and Man‐C6‐SS‐NPs, 0.2 μg/mL) were added and incubated in the incubator for 0.5 and 1.5 h, respectively. After the incubation, the following operations were performed according to 3.6.1. Finally, the cell morphology and the uptake of the preparation were observed under a laser confocal microscope.

#### 3.8.2 The uptake of nanoparticles in MDA‐MB‐231 cells was quantitatively investigated

UPLC‐MS/MS was used to determine the uptake of PTX, ProPTX, and prodrug nanoparticles in MDA‐MB‐231 cells at different concentrations and at different times. First, MDA‐MB‐231 cells were seeded into 24‐well culture plates at a density of 1 × 10^5^ cells/mL for 24 h, and 1 mL was seeded into each well. After cell adhesion, the culture medium was discarded and the cell layer was washed twice with the HBSS solution. To investigate the relationship between drug uptake and time, MDA‐MB‐231 cells were treated with 5 μM PTX, ProPTX, ProPTX‐NPs, ProPTX‐SS‐NPs, and Man‐ProPTX‐SS‐NPs solutions (measured by paclitaxel concentration) for 1 and 2 h, respectively. To investigate the relationship between drug uptake and concentration, MDA‐MB‐231 cells were incubated with different concentrations (1 μM, 5, and 10 μM) for 2 h. At the end of incubation, the liquid was discarded and washed with PBS for 3 times. Then, 200 μL 0.1% TritonX‐100 PBS solution was added to the cells and stood for 30 min to lysate the cells. Then, the cells were scraped off the culture plate and suspended in a 1.5 mL centrifuge tube. Q‐trap was used to quantitatively investigate the uptake of the cells.

#### 3.8.3 Study of the uptake mechanism

To investigate the mechanism of Man‐C6‐SS‐NPs uptake by MDA‐MB‐231 cells, flow cytometry was used to investigate the effects of various endocytic inhibitors (sodium azide, chlorpromazine, indomethacin, *β*‐cyclodextrin, quercetin, and low temperature) on the uptake process. Chlorpromazine can prevent clathrin and AP‐2 protein complex from migrating from the cell membrane to the primary endocytosis, thereby inhibiting clathrin‐mediated endocytosis [14]. Sodium azide is an energy inhibitor, which can inhibit the activity of cytochrome C oxidase in the mitochondrial electron transport chain [15]. Similar to the low‐temperature (4°C) environment, sodium azide can affect cellular uptake by inhibiting the generation of intracellular ATP. Indomethacin is a COX/caveolin‐mediated endocytosis inhibitor [16, 17]. Quercetin is a non‐clathrin and non‐caveolin‐mediated endocytosis inhibitor [18]. *β*‐cyclodextrin is a lipid raft/caveolin‐mediated endocytosis inhibitor [19].

### 3.9 Receptor affinity

To investigate the affinity of Man‐C6‐SS‐NPs with the CD206 receptor on the surface of tumour cells, we used MDA‐MB‐231 cells with a high expression of CD206 receptor as a model [20, 21] and selected free mannose as a competitive inhibitor of the CD206 receptor. The competitive inhibition of mannose on the uptake of Man‐C6‐SS‐NPs nanoparticles was investigated quantitatively and qualitatively. Free mannose can specifically bind to the CD206 receptor on MDA‐MB‐231 cells [22, 23].

#### 3.9.1 Qualitative examination

The affinity of C6‐Sol, C6‐NPs, C6‐SS‐NPs, and Man‐C6‐SS‐NPs to CD206 receptor was qualitatively investigated by CLSM. MDA‐MB‐231 cells were seeded into laser confocal culture dishes at the density of 1 × 10^5^ cells/mL and cultured for 24 h. After adherent growth of cells, the administration protocol was the same as 2.8.1, the uptake of cells was terminated with PBS at 4°C, and the other operations were the same as described in Section 3.8.1. Confocal laser microscopy was used to investigate the targeting situation.

#### 3.9.2 Quantitative examination

The affinity of C6‐Sol, C6‐NPs, C6‐SS‐NPs, and Man‐C6‐SS‐NPs to CD206 receptor was quantified by flow cytometry. First, MDA‐MB‐231 cells were seeded into 12‐well plates at a density of 1.5 × 10^5^ cells/mL and cultured for 24 h. After adherent growth, the culture medium was discarded and washed twice with a serum‐free medium, then the blank medium and the mannose solution (5 mg/mL) were added and preincubated for 1 h. The solution in the culture Wells was discarded, and C6‐Sol, C6‐NPs, C6‐SS‐NPs, and Man‐C6‐SS‐NPs were added to the blank Wells, respectively. Man‐C6‐SS‐NPs was added to the mannose solution treatment Wells, and the incubation was continued for 1 h. The other operations were the same as described in Section 3.8.2, and the targeting was quantitatively investigated by flow cytometry.

## 4 RESULTS AND DISCUSSIONS

### 4.1 Pharmacologic characteristics of nanoparticles

**FIGURE 1:**
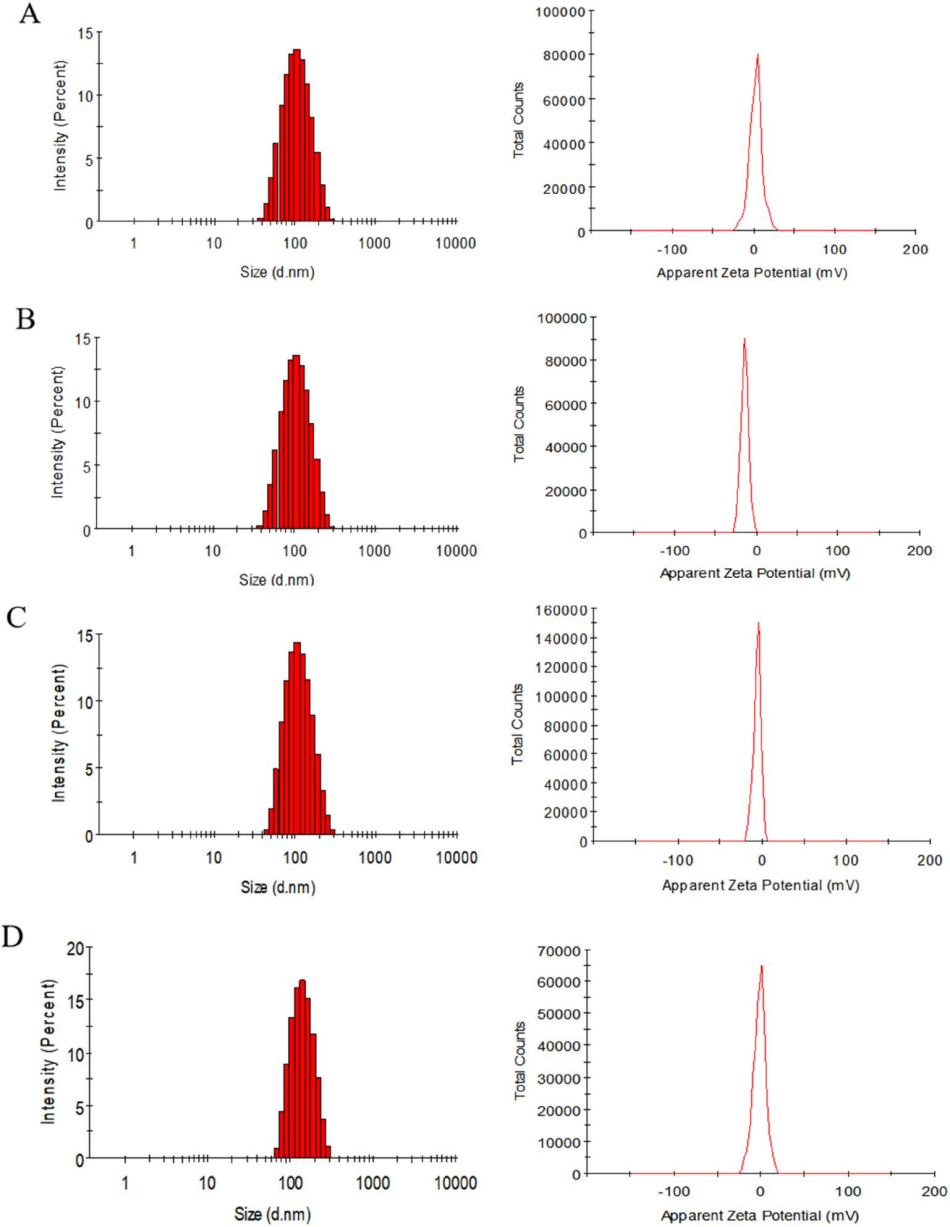
The particle size and potential of Blank‐PP‐NPs. (a) ProPTX‐NPs (b) ProPTX‐SS‐NPs (c), and Man‐ProPTX‐SS‐NPs (d) (Mean ± SD, *n* = 3).

The particle size and distribution of nanoparticles are important factors affecting the physical stability of the particle dispersion system [24]. The particle sizes of the four paclitaxel prodrug nanoparticles were uniform, all around 130 nm, which met the EPR effect size (Figure 1). The Zeta potential is about −8 mV, which can make the nanoparticles avoid aggregation through the charge repulsion on the surface, thus improving the stability. The encapsulation rate (EE) and DL of nanoparticles were 95.46 ± 1.57 and 13.65 ± 2.31, respectively (Table 1), which met the standards of nanoparticles.

**TABLE 1:** Characterisation of nanoparticles (Mean ± SD, *n* = 3).

Nanoparticle	Size (nm)	PDI	Zeta (mV)	EE (%)	DL (%)
Blank‐PP‐NPs	110.24 ± 3.57	0.27 ± 0.02	– 6.47 ± 0.01	–	–
ProPTX‐NPs	116.60 ± 1.57	0.13 ± 0.03	– 7.20 ± 0.70	94.01 ± 3.41	12.57 ± 1.07
ProPTX‐SS‐NPs	130.20 ± 2.18	0.12 ± 0.01	– 8.45 ± 0.01	93.22 ± 2.20	10.27 ± 1.36
Man‐ProPTX‐SS‐NPs	132.90 ± 1.81	0.13 ± 0.03	– 8.65 ± 0.50	95.46 ± 1.57	13.65 ± 2.31

#### 4.1.1 Colloidal stability

**FIGURE 2:**
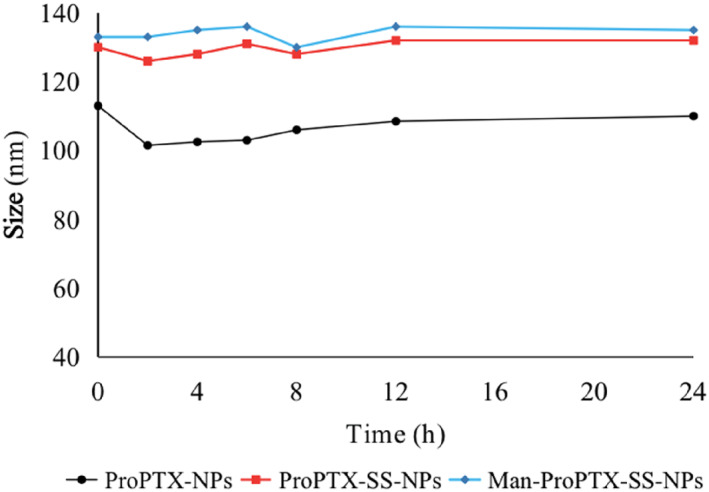
Particle size changes of ProPTX‐NPs and ProPTX‐SS‐NPs nanoparticles in PBS containing 10% foetal bovine serum (pH 7.4) (Mean ± SD, *n* = 3).

When the two prodrug nanoparticles were co‐incubated with the incubation medium, the particle size of both nanoparticles decreased slightly at 2 h, but at 4 h, the particle size of the two nanoparticles increased and stabilised, similar to that of the original preparation, and remained unchanged at the following 20 h (Figure 2). It indicates that BSA can be adsorbed on the surface of nanoparticles and acts as a stabilizer, making the structure of nanoparticles more compact and providing better colloidal stability. The results verified that the two prodrug nanoparticles had good colloidal stability under simulated physiological conditions, which could be used for subsequent studies.

### 4.2 Cytotoxicity

**FIGURE 3:**
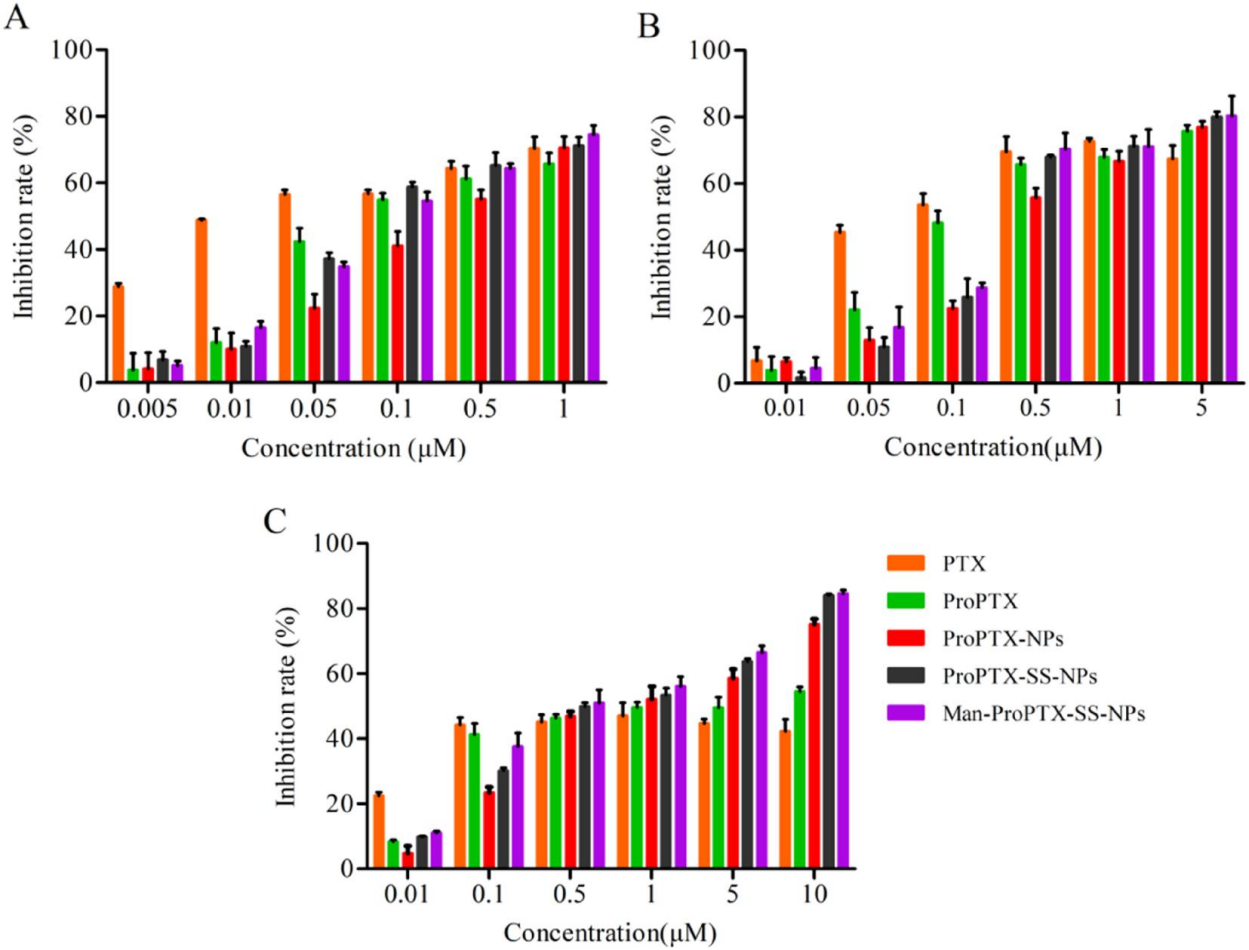
Cytotoxicity of PTX, ProPTX, ProPTX‐NPs, ProPTX‐SS‐NPs, and Man‐ProPTX‐SS‐NPs after 48 h incubation with MCF‐7 (a), HepG2 (b), and MDA‐MB‐231 (c) cells (mean ± SD, *n* = 3).

MTT assay was used to investigate the cytotoxicity of PTX, ProPTX, and three kinds of nanoparticles co‐incubated with MCF‐7, HepG2, and MDA‐MB‐231 cells for 48 h, respectively. In MCF‐7 and HepG2 cells, PTX, ProPTX, and the three nanoparticles showed strong cytotoxicity in a concentration‐dependent manner (Figure 3a,b). On MDA‐MB‐231 cells, from 0.1 to 10 μm, the tumour suppressive effect of the prodrug solution and nanoparticles was higher than that of PTX, and the tumour suppressive effect of prodrug nanoparticles was higher than that of the prodrug solution (Figure 2C).

The toxicity of each nanoparticle to MDA‐MB‐231 cells from small to large was: ProPTX‐NPs < ProPTX‐SS‐NPs < Man‐ProPTX‐SS‐NPs, IC_50_ was: 1.26 μM < 0.71 μM <0.52 μM (Table 2). The cytotoxicity of ProPTX‐NPs was compared to ProPTX and lower than that of Man‐ProPTX‐SS‐NPs and ProPTX‐SS‐NPs. These results indicate that Man‐ProPTX‐SS‐NPs and ProPTX‐SS‐NPs have GSH sensitivity and can release drugs rapidly under the condition of a high concentration of GSH in tumour cells. However, the toxicity of the targeted agent Man‐ProPTX‐SS‐NPs was significantly higher than that of the non‐targeted agent ProPTX‐SS‐NPs, because the CD206 receptor overexpressed on MDA‐MB‐231 cells can bind specifically to mannose on the surface of Man‐ProPTX‐SS‐NPs, thereby increasing the accumulation of the agent in the tumour cells.

**TABLE 2:** IC_50_ values of PTX, ProPTX, and drug‐loaded nanoparticles incubated with MCF‐7, HepG2, and MDA‐MB‐231 cells for 48 h, respectively.

Formulations	IC_50_ (μM)		
	MCF‐7	HepG2	MDA‐MB‐231
PTX	0.02 ± 0.01	0.09 ± 0.01	22.38 ± 3.27
ProPTX	0.08 ± 0.12***	0.18 ± 0.01***	1.23 ± 0.32***
ProPTX‐NPs	0.16 ± 0.03	0.39 ± 0.03	1.26 ± 0.60
ProPTX‐SS‐NPs	0.08 ± 0.01	0.24 ± 0.13	0.71 ± 0.11
Man‐ProPTX‐SS‐NPs	0.07 ± 0.12***	0.21 ± 0.01***	0.52 ± 0.12***^,^^###^

(Mean ± SD, *n* = 3) (Different nanoparticles in the same cell line: ***P < 0.001, vs. PTX; ^###^ *P* < 0.001, vs. ProPTX‐SS‐NPs).

Notably, the activity of ProPTX was lower than that of PTX in the cytotoxicity assay. This is mainly because after the prodrug enters the cell, it needs to release paclitaxel under the action of ROS to play an anti‐tumour effect. However, intracellular ROS is not enough to convert all ProPTX into PTX, so the activity of the prodrug is less than that of PTX. Compared with ProPTX, ProPTX‐NPs are less effective than ProPTX due to slow drug release. However, Man‐ProPTX‐SS‐NPs, a sensitive and targeted agent, and ProPTX‐SS‐NPs, a sensitive and non‐targeted agent, can react with GSH in tumour cells and rapidly release the drug. Therefore, the toxicity of Man‐ProPTX‐SS‐NPs is compared to that of ProPTX, which is higher than that of ProPTX‐NPs. In addition, the cytotoxicity of Man‐ProPTX‐SS‐NPs and ProPTX‐SS‐NPs was not significantly different because neither MCF‐7 nor HepG2 cells were CD206 receptor overexpressing cells.

### 4.3 Apoptosis

**FIGURE 4:**
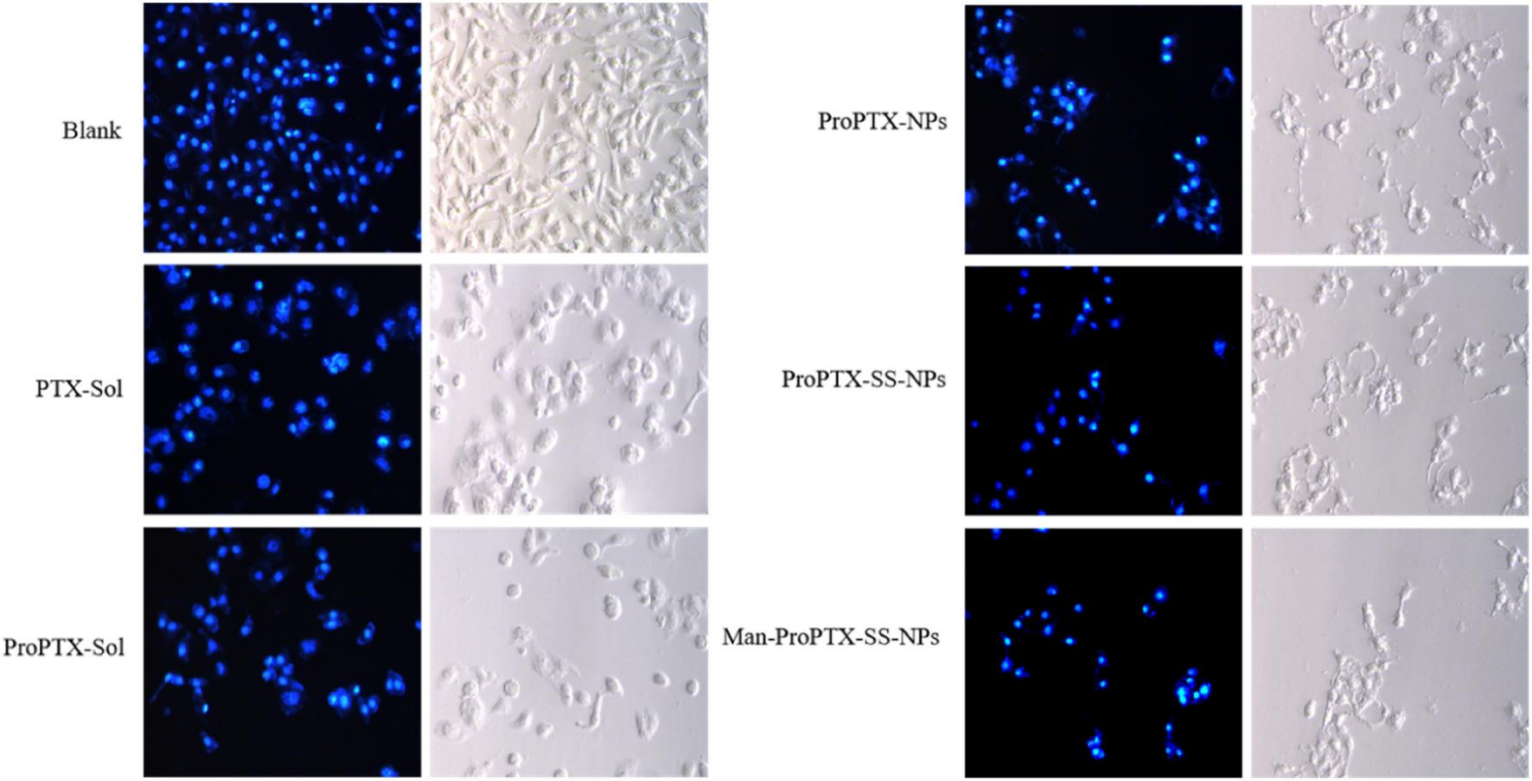
Cell morphology of PTX, ProPTX, ProPTX‐NPs, ProPTX‐SS‐NPs, and Man‐proPTX‐SS‐NPs after 48 h incubation with MDA‐MB‐231 cells. The magnification was 60×. Scale bar was 50 μm.

The morphological changes of MDA‐MB‐231 cells treated with each preparation for 48 h were observed by an inverted fluorescence microscope. MDA‐MB‐231 cells were treated with PTX, ProPTX, and three kinds of nanoparticles, and the number of viable cells decreased significantly. The viable cell survival was PTX > ProPTX > ProPTX‐NPs > ProPTX‐SS‐NPs > Man‐ProPTX‐SS‐NPs, which was consistent with the cytotoxicity results (Figure 4). At the same time, after PTX and ProPTX treatment, the cell membrane boundary became round and the morphology became larger. After the treatment of ProPTX‐NPs, ProPTX‐SS‐NPs, and Man‐ProPTX‐SS‐NPs, the cell debris gradually increased, the cell membrane significantly wrinkled, the boundary became blurred, the cell morphology and integrity were damaged, and the cell apoptosis occurred. Therefore, it is speculated that paclitaxel induces the death of MDA‐MB‐231 breast cancer cells by first inducing cell expansion, then causing cell rupture, and finally gradually lytic cells [24]. PTX and ProPTX treatment made the cells swell. However, ProPTX‐NPs, ProPTX‐SS‐NPs, and Man‐ProPTX‐SS‐NPs made the cells in the state of rupture and lysis after co‐incubation with MDA‐MB‐231 cells because of the greater cytotoxicity. Therefore, it can be seen that prodrug nanoparticles can better induce apoptosis at the same dose.

#### 4.3.1 Detection of apoptosis with the apoptosis kit

**FIGURE 5:**
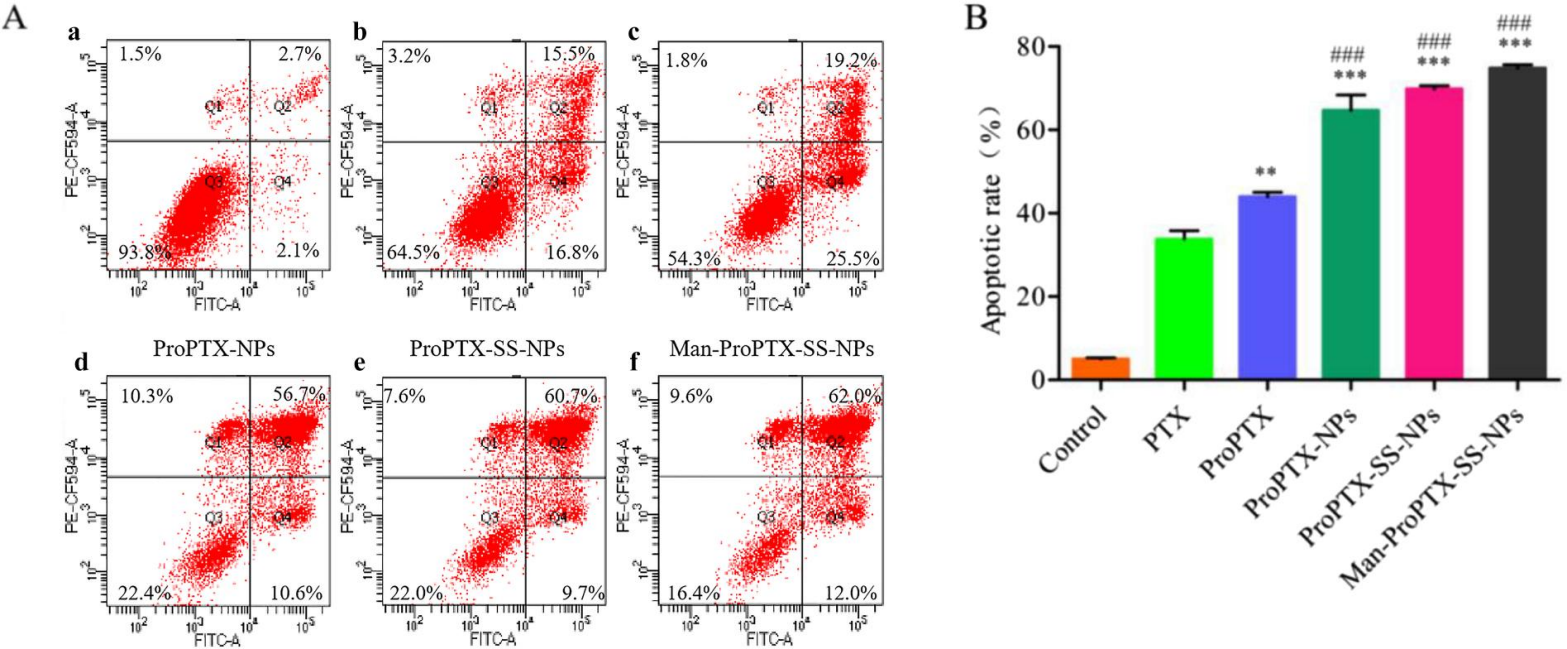
Comparison of 48 h apoptosis results of Control (a), PTX (b), ProPTX (c), ProPTX‐NPs (d), ProPTX‐SS‐NPs (e), and Man‐ProPTX‐SS‐NPs (f) on MDA‐MB‐231 cells (a). Histogram analysis of the apoptosis rate (b) (Mean ± SD, *n* = 3) (**P < 0.01, ***P < 0.001, vs. PTX, ^###^ *P* < 0 0.001 vs. ProPTX).

The apoptosis effect of the three nanoparticles on MDA‐MB‐231 cells was significantly higher than that of ProPTX and PTX (*P* < 0.05). In MDA‐MB‐231 cells treated with the three nanoparticles for 48 h, the number of early apoptotic cells was significantly lower than that of ProPTX and PTX, but the number of late apoptotic cells increased by 30%–45% compared with ProPTX and PTX. The number of necrotic cells also increased by 4.4%–8.5%. These results indicated that at the same concentration, nanoparticles could induce apoptosis more significantly than solution agents (Figure 5a).

The paclitaxel solution and all preparation groups could induce apoptosis significantly. Compared with PTX, ProPTX significantly increased the apoptosis rate of MDA‐MB‐231 cells (*P* < 0.001), which was consistent with the cytotoxicity results (Figure 5b). This is because ProPTX can increase the intracellular PTX concentration through increased uptake mediated by sialic acid receptors. After being treated with Man‐ProPTX‐SS‐NPs, ProPTX‐SS‐NPs, and ProPTX‐NPs for 48 h, the apoptosis rates of MDA‐MB‐231 cells were 74.95%, 69.80%, and 64.70%, respectively, which were significantly higher than those of ProPTX (the apoptosis rate was 43.95%). This was consistent with the results of a qualitative investigation of cell apoptosis, and Man‐ProPTX‐SS‐NPs had a more significant effect on inducing cell apoptosis. These results again confirmed that Man‐ProPTX‐SS‐NPs with GSH sensitivity and CD206 targeting had a better antitumour effect.

### 4.4 Detection of ROS

**FIGURE 6:**
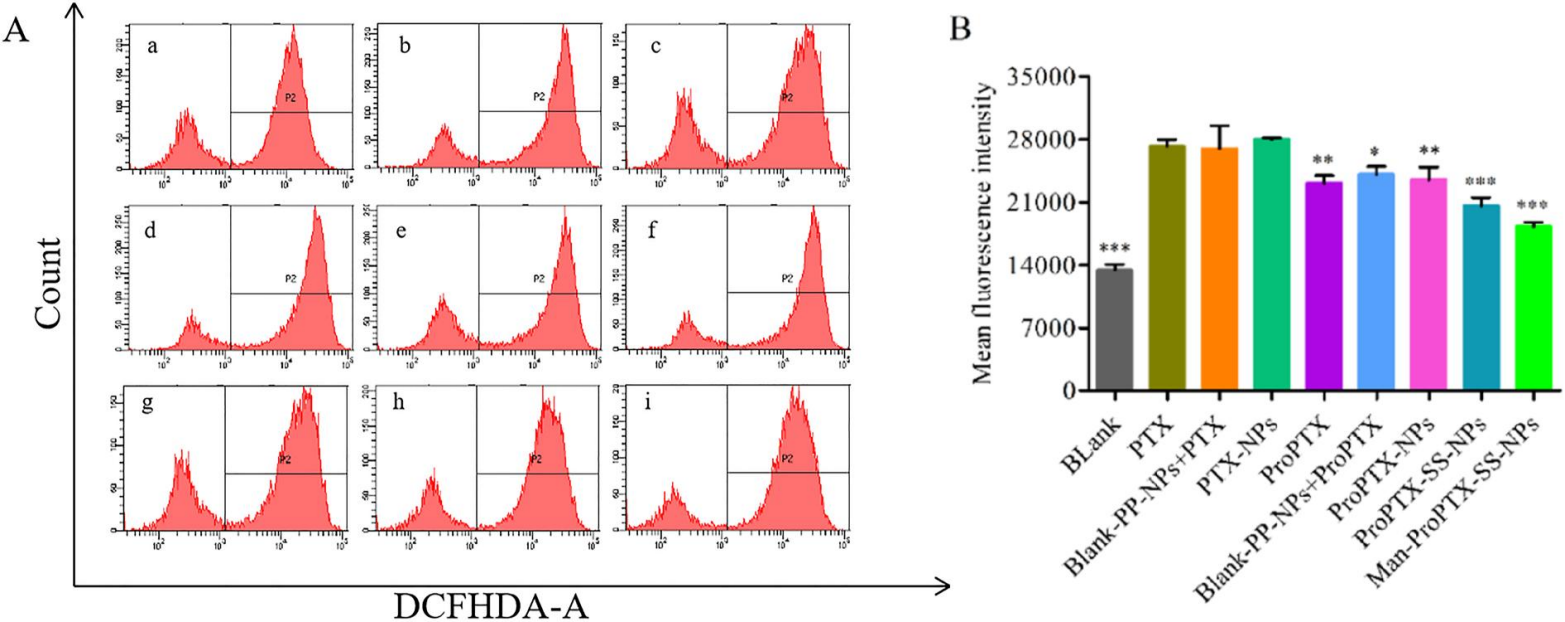
Blank (PBS Sterile) (a), PTX (b), Blank PP‐NPs and PTX physical mixture (c), PTX‐NPs (d), ProPTX (e), Blank PP‐NPs and ProPTX physical mixture (f), ProPTX‐NPs (g), ProPTX‐SS‐NPs (h), and Man‐ProPTX‐SS‐NPs (i) in MDA‐MB‐21 reactive oxygen species (ROS) determination (a). Statistical analysis of test results (b) (Mean ± SD, *n* = 3) (*P < 0.05, **P < 0.01, ***P < 0.001, vs. PTX).

MDA‐MB‐231 cells were treated with paclitaxel, paclitaxel prodrugs, and their preparations, and the intracellular ROS levels were significantly increased. This is because paclitaxel itself increases the level of ROS in tumour cells [25, 26]. The levels of ROS in ProPTX, Blank‐PP‐NPs, and ProPTX physical mixture groups were significantly decreased (*P* < 0.05), which indicated that the prodrug needed to consume H_2_O_2_ to release PTX and was sensitive to H_2_O_2_ (Figure 6). There was no difference in ROS levels between Blank‐PP‐NPs, PTX‐NPs, and Blank‐PP‐NPs and PTX groups, indicating that Blank nanoparticles did not consume or produce ROS. The ROS of ProPTX‐SS‐NPs and Man‐ProPTX‐SS‐NPs were lower, which were due to the high level of GSH in tumour cells. The disulfide bond in the sensitive preparations was easily broken, which accelerated the release of ProPTX and consumed more ROS. The targeted agent Man‐ProPTX‐SS‐NPs consumed the most ROS. This is because it can mediate more ProPTX intake via CD206 and therefore consume more ROS. This also further verified the mannose targeting and GSH sensitivity of the constructed Man‐ProPTX‐SS‐NPs preparation on MDA‐MB‐231 cells.

### 4.5 Cell uptake

#### 4.5.1 Fluorescence microscope observation

**FIGURE 7:**
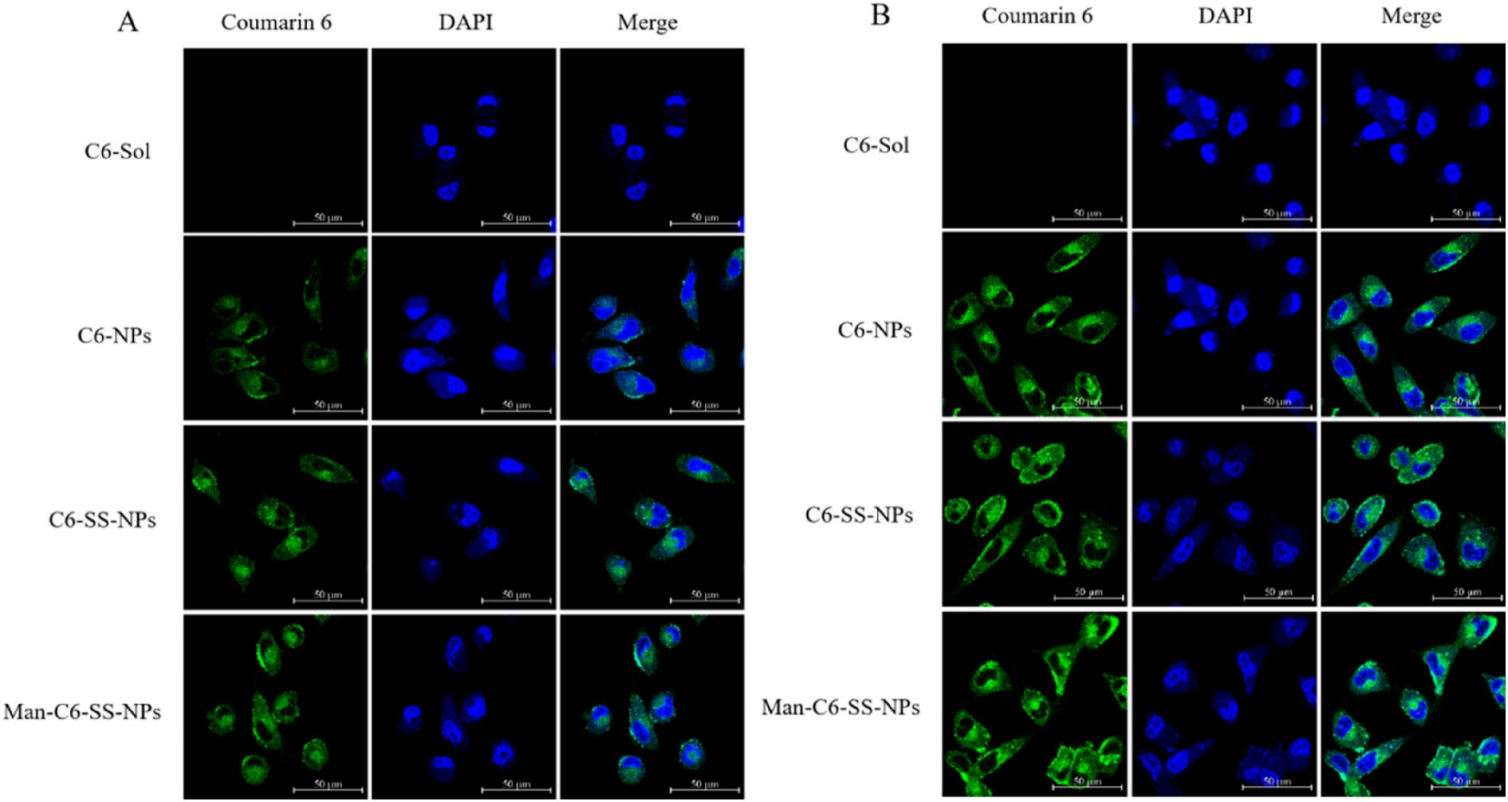
The uptake of C6‐Sol and C6 nanoparticles on MDA‐MB‐231 cells at 0.5 h (a) and 1.5 h (b) (Mean ± SD, *n* = 3) was observed by the confocal laser microscopy. The magnification was 60×. Scale bar was 50 μm.

The cells treated with C6 nanoparticles were in good condition, and no obvious cell membrane shrinkage was observed in all preparation groups (Figure 7). The fluorescence was mainly concentrated in the cytoplasm, and the fluorescence intensity increased significantly with the increase of incubation time, indicating that the uptake of different nanoparticles was time‐dependent. The fluorescence intensity of the three C6 nanoparticles was significantly stronger than that of C6‐Sol, indicating that the nanoparticles had higher cellular uptake efficiency than the solution form. This may be because nanoparticles enter cells through an endocytic pathway, which avoids the recognition of efflux proteins to a certain extent. In addition, the fluorescence signal of Man‐C6‐SS‐NPs was stronger than that of C6‐SS‐NPs due to its CD206 targeting to increase the cellular uptake.

#### 4.5.2 Relationship between intake and intake time

**FIGURE 8:**
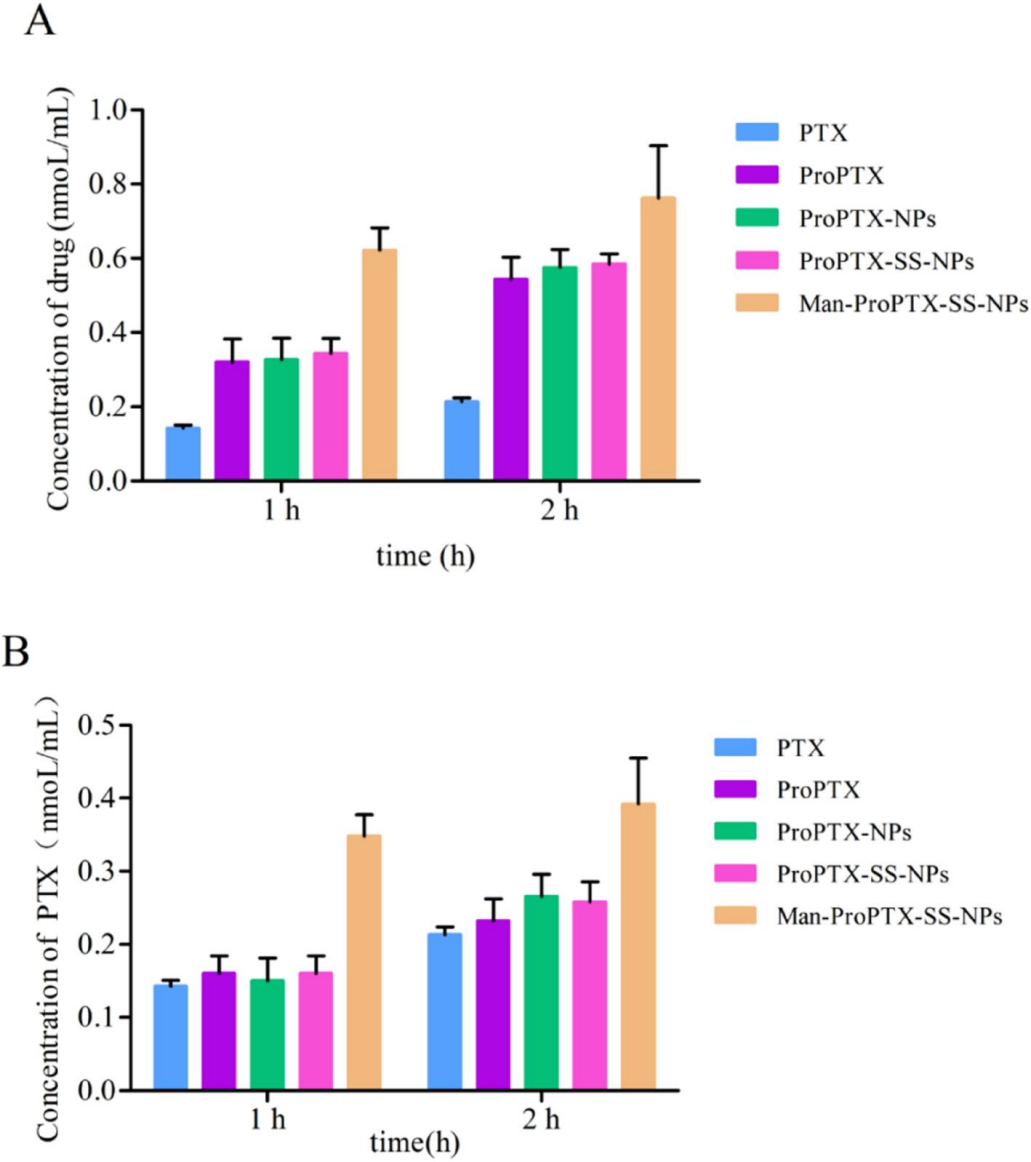
The consumption of paclitaxel (paclitaxel prodrug + paclitaxel) (a) and the drug alone (paclitaxel) (b) in MDA‐MB‐231 cells as a function of time (mean ± SD, *n* = 3).

The uptake of PTX, ProPTX, and the three nanoparticles by MDA‐MB‐231 cells was time‐dependent and increased with the extension of uptake time. Both at 1 and 2 h, the order of cell intake of each drug group was Man‐ProPTX‐SS‐NPs > ProPTX‐SS‐NPs ≈ ProPTX‐NPs ≈ ProPTX > PTX (Figure 8a). Compared with PTX, ProPTX can be actively transported into cells mediated by sialic acid on the cell surface, which avoids cell efflux, thus significantly increasing the cellular uptake of the drug. The uptake of ProPTX‐SS‐NPs, ProPTX‐NPs, and ProPTX was similar, indicating that the introduction of disulfide bonds in the drug loading system did not affect the uptake of the preparation in cells. The uptake of Man‐ProPTX‐SS‐NPs was the highest, indicating that the modification of mannose targeting CD206 by nanoparticles could bind to the overexpressed CD206 receptor on MDA‐MB‐231 cells and improve the drug uptake. After incubation of MDA‐MB‐231 cells with ProPTX and three‐drug nanoparticles, nearly half of the paclitaxel prodrug released by the preparation after entering the cells was degraded to paclitaxel, indicating that the ROS in tumour cells can hydrolyse the paclitaxel prodrug to the parent drug (Figure 8b).

#### 4.5.3 Relationship between intake and drug concentration

**FIGURE 9:**
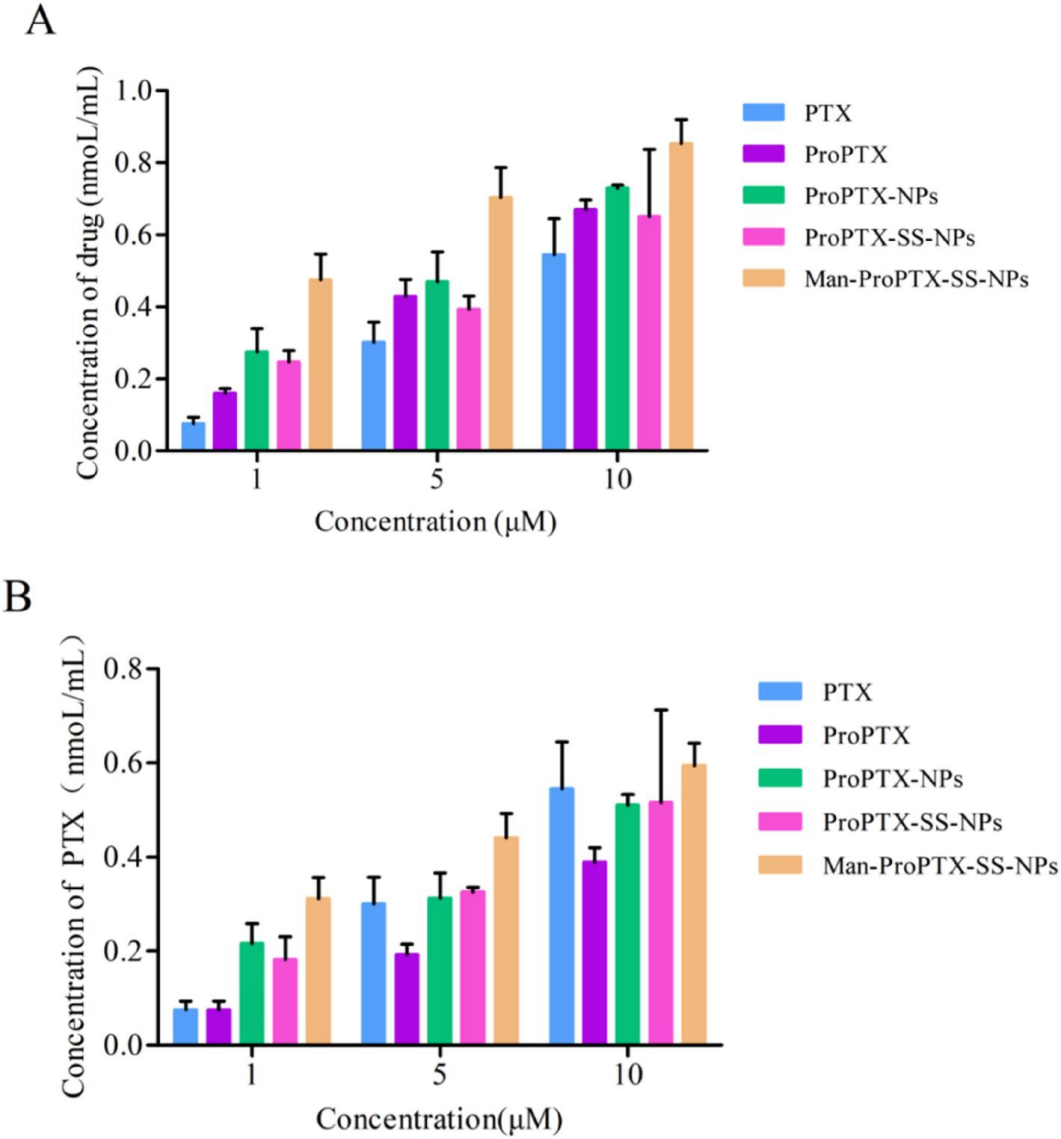
Total drug (paclitaxel prodrug + paclitaxel) (a) and drug alone (paclitaxel) (b) intake curves of paclitaxel and each nanoparticle on MDA‐MB‐231 cells as a function of concentration (mean ± SD, *n* = 3).

The drug uptake by MDA‐MB‐231 cells increased with the increase of drug concentration, indicating that the drug uptake by cells was concentration‐dependent to some extent (Figure 9a). At the concentration of 1 μM, 5 μM, or 10 μM, the intake was Man‐ProPTX‐SS‐NPs > ProPTX‐SS‐NPs ≈ ProPTX‐NPs. This was consistent with the results of the relationship between cell uptake and time, which again verified the GSH sensitivity of ProPTX‐SS‐NPs and Man‐ProPTX‐SS‐NPs and the CD206 receptor targeting of Man‐ProPTX‐SS‐NPs. Nanoparticles can release free PTX after uptake into cells. However, the paclitaxel content of PTX was higher than that of ProPTX and the three prodrug nanoparticles at high concentrations. This is because the level of ROS in the cells at this time is not sufficient to reduce the entire paclitaxel prodrug to paclitaxel in a short time, resulting in a low paclitaxel content in ProPTX and the three prodrug nanoparticle solutions (Figure 9b). The targeted Man‐ProPTX‐SS‐NPs, by modifying the mannose to increase drug uptake, released more PTX even in a short period.

#### 4.5.4 The mechanism of cell uptake

**FIGURE 10:**
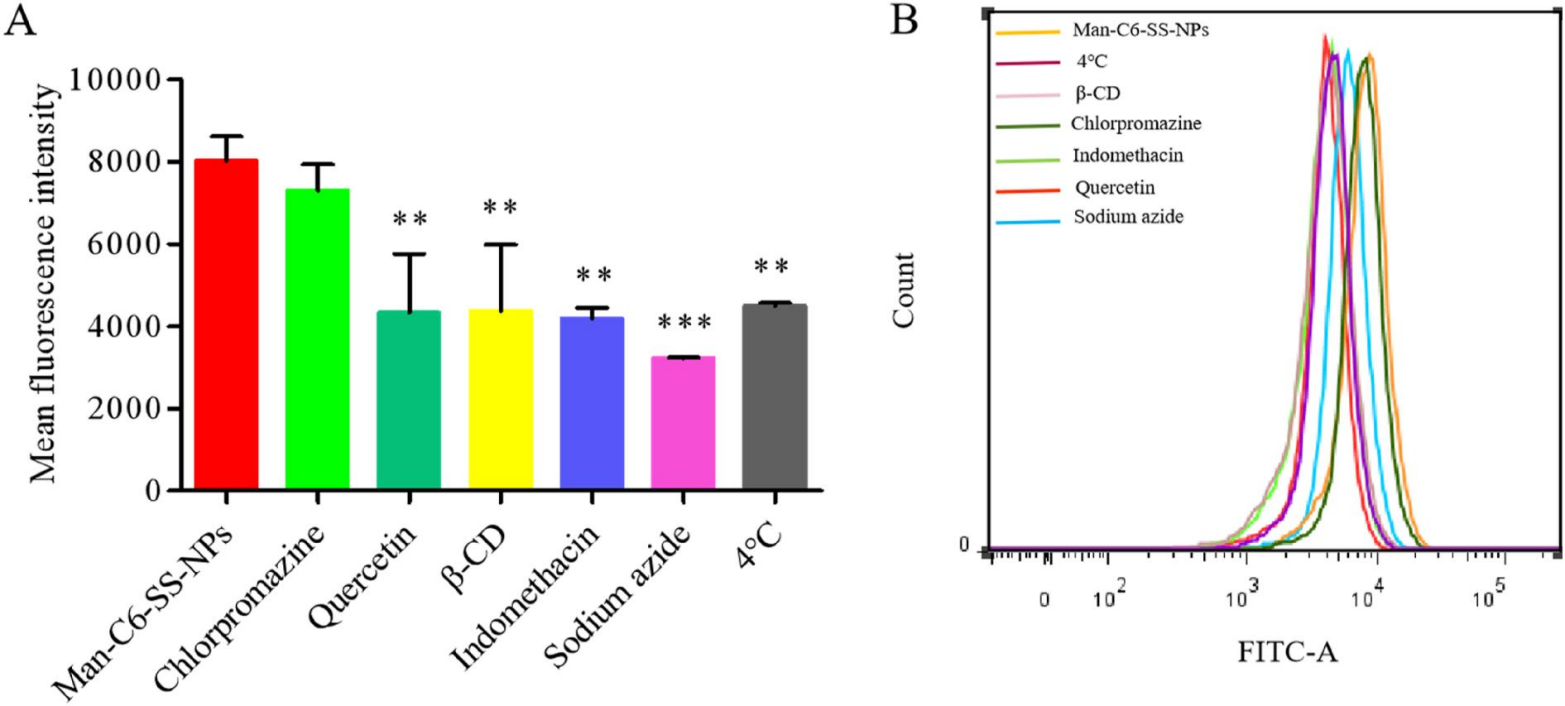
(a and b) The effect of various endocytic inhibitors on the uptake of Man‐C6‐SS‐NPs in MDA‐MB‐231 cells was quantified by flow cytometry (mean ± SD, *n* = 3) (*P < 0.05, vs. Man‐C6‐SS‐NPs).

Compared with the blank control group, the intake of MDA‐MB‐231 cells was not significantly changed after chlorpromazine addition, indicating that the uptake mechanism of nanoparticles was not mediated by clathrin and AP‐2 protein complex. However, Quercetin, *β*‐cyclodextrin, and Indomethacin all significantly inhibited the drug uptake in cells (*P* < 0.05),indicating that Man‐C6‐SS‐NPs could enter cells through endocytosis mediated by non‐clathrin, non‐caveolin, lipid raft/caveolin, and cyclooxygenase (COX)/caveolin. In addition, sodium azide and low temperature (4°C) also significantly inhibited the uptake of nanoparticles by cells (*P* < 0.05), indicating that the uptake of Man‐C6‐SS‐NPs by MDA‐MB‐231 cells requires energy. In conclusion, the mechanism of cellular uptake is energy‐dependent and co‐mediated by non‐clathrin, non‐caveolin, lipid raft/caveolin, and cycidase (COX)/caveolin endocytosis (Figure 10).

### 4.6 Receptor affinity

**FIGURE 11:**
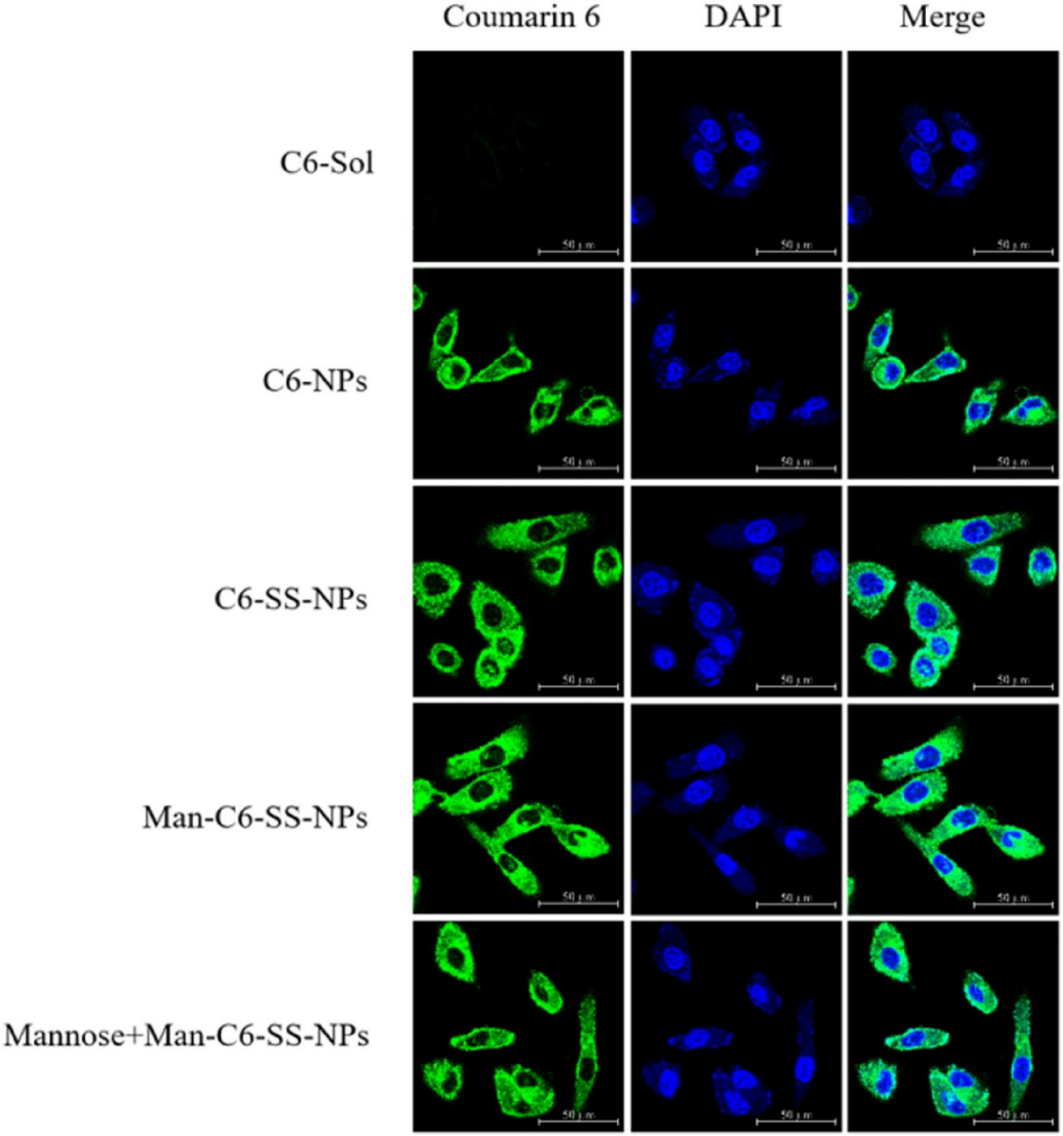
Laser confocal images of C6‐Sol and C6 nanoparticles incubated with MDA‐MB‐231 cells for 1.5 h (Mean ± SD, *n* = 3) magnification: 60×. Scale bar: 50 μm.

**FIGURE 12:**
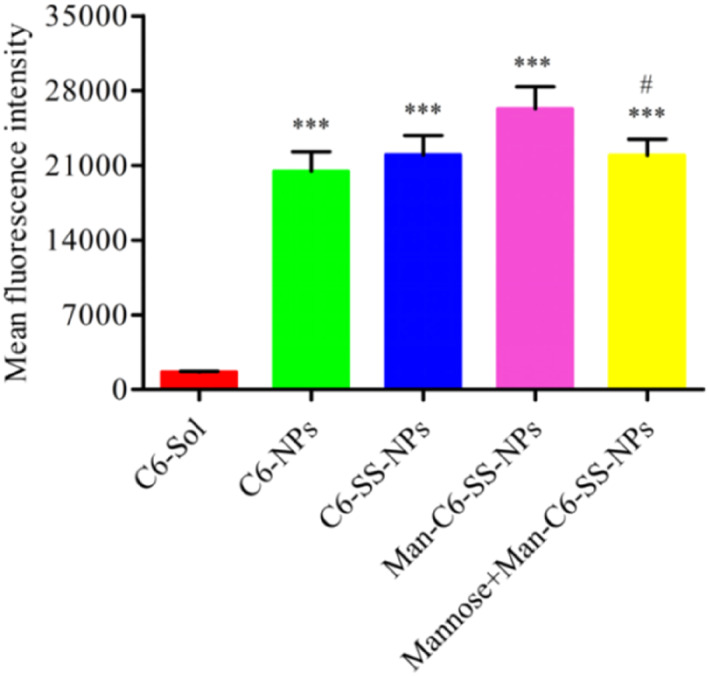
The uptake of C6‐Sol in MDA‐MB‐231 cells after 2 h incubation was quantified by flow cytometry (mean ± SD, *n* = 3) (***P < 0.001 vs. C6‐Sol, ^#^ *P* <   0.05 vs. Man‐C6‐SS‐NPs).

Compared with the solution agent, all drug‐loaded nanoparticles could significantly increase the accumulation of coumarin‐6 in MDA‐MB‐231 cells (Figure 11). Compared with C6‐SS‐NPs, the fluorescence intensity of Man‐C6‐SS‐NPs was higher. After adding the mannose solution, the fluorescence intensity of the Man‐C6‐SS‐NPs group was significantly decreased. These results suggested that Man‐C6‐SS‐NPs could bind to the overexpressed CD206 receptor on tumour cells and increase the uptake of the preparation, which confirmed the CD206 receptor targeting of Man‐C6‐SS‐NPs. This conclusion has been more fully proved in our further quantitative study (Figure 12).

## 5 CONCLUSION

Paclitaxel, with its remarkable antitumour activity and novel mechanism of action, became a famous anticancer star and research focus in the second half of the 20th century. However, paclitaxel is not selective for tumour tissue. After intravenous injection, it is distributed to tumour tissue to kill tumour cells and also to normal tissue to damage normal cells, which is the main reason for adverse reactions. At present, there are some clinical preparations used to improve the antitumour efficacy of paclitaxel, such as Taxol^®^, which aims to improve solubility, liposomal (e.g. Lipusum^®^, LEP‐ETU^®^) and micellar type (e.g. Genexol‐PM^®^, Nanoxel^®^) preparations to prolong drug circulation in vivo and reduce drug distribution in normal tissues, as well as albumin solvent‐based nanopaclitaxel (ABI‐007) and liposome (Ceptin) with active targeting [27]. However, the targeting efficiency of these agents is difficult to be satisfactory, and the distribution of paclitaxel in normal tissue sites is still difficult to avoid, which largely limits its further clinical applications [28].

In our research, a targeted nano delivery system Man‐PEG‐SS‐PLGA/Pro‐PTX for tumour microenvironment response was constructed in this study. In order to limit the biological activity of drugs in normal tissues, we constructed a preparation that can only release active drugs under the dual stimulation of high concentration of GSH and ROS. However, due to the low concentration of GSH and ROS in normal tissues, the drug activity is blocked, so it can only be distributed to the tumour site through the circulation in vivo or excreted through the circulation. It was proved that this nano‐delivery system modified with the Mannose structure could selectively deliver the PTX system to tumour sites and increase the accumulation of drugs in tumour sites, which is conducive to further reducing the drug toxicity of paclitaxel. On the other hand, Mannose is a natural monosaccharide in fruits. PEG [28] and PLGA [29] have good biocompatibility and can be degraded into non‐toxic substances in the body. At present, both of them are approved by FDA for medicinal use. The nano‐delivery system constructed with these materials has no special toxic substances, which provides favourable conditions for its further clinical application.

Admittedly, this conclusion still needs to be further verified in animal models, but it is undeniable that Man‐PEG‐SS‐PLGA/Pro‐PTX is a promising tumour microenvironment‐responsive targeted nano delivery system, which is expected to solve the current clinical limitations of PTX and provide new ideas and approaches for clinical application of PTX.

## AUTHOR CONTRIBUTIONS

**Changhai Wang**: Formal Analysis, Methodology, Writing ‐Original Draft. **Yuwen Jiao & Xinyu Zhang**: Data curation, Investigation. **Xinyu Zhang**: Writing & Review & Editing. **Mingxue Guo**: Validation. **Wenjun Hu**: Data Curation, Investigation. **Wenjun Hu & Tangthianchaichana Jakkree**: Validation. **Jinling Wang**: Funding Acquisition, Conceptualization. **Yang Lu**: Conceptualization, Resources.

## CONFLICT OF INTEREST STATEMENT

The authors declare that they have no competing interests.

## Data Availability

Data generated during the study are subject to a data sharing mandate and available in a public repository that does not issue datasets with DOIs.
